# The Microbiome–Metabolome Response in the Colon of Piglets Under the Status of Weaning Stress

**DOI:** 10.3389/fmicb.2020.02055

**Published:** 2020-08-28

**Authors:** Xueyuan Jiang, Naisheng Lu, Haichao Zhao, Hao Yuan, Dong Xia, Hulong Lei

**Affiliations:** ^1^Institute of Animal Husbandry and Veterinary Science, Shanghai Engineering Research Center of Breeding Pig, Shanghai Academy of Agricultural Sciences, Shanghai, China; ^2^Department of Pharmaceutical Microbiology, School of Life Sciences and Technology, China Pharmaceutical University, Nanjing, China

**Keywords:** piglets, weaning stress, cortisol, microbiome, metabolome

## Abstract

Weaning is stressful for piglets involving nutritional, physiological, and psychological challenges, leading to an increase in the secretion of cortisol, changes in gut microbiome and metabolites, whereas the underlying relationships remain unclear. To elucidate this, 14 Meishan female piglets were divided into the weaning group and the suckling group at the age of 21 days paired by litter and body weight. After 48 h of experiment, weaned piglets had lower body weight, but higher salivary cortisol level than that of their suckling litter mates (*P* < 0.05). The composition of the colonic bacterial community and metabolites were different between the two groups, and the first predominant genus of the suckling and weaned piglets colonic microbiome were *Bacteroides* and *Prevotellaceae-NK3B31 group* respectively. The suckling piglets had higher proportions of phylum *Bacteroidetes* and *Lentisphaerae*, and genus *Bacteroides* and *Lactobacillus* in the colonic microbial community, but lower abundance of genus *Prevotellaceae-NK3B31 group* than that of the weaned piglets (*P* < 0.05). Accordingly, there were 15 colonic metabolites differed between the two groups, in which 2 metabolites (phenylacetic acid and phenol) negatively related to the abundant of *Lactobacillus* genus (*P* < 0.05), while 9 metabolites (acetic acid, arabitol, benzoic acid, caprylic acid, cholesterol, dihydrocholesterol, galactinol, glucose phenol, phenylacetic acid, and oxamic acid, glycerol, propionic acid) positively associated with the proportion of *Prevotellaceae-NK3B31 group* genus (*P* < 0.05). Furthermore, the salivary cortisol level negatively associated with the abundance of phylum *Lentisphaerae*, but positively associated with the phylum *Bacteroidetes* and the genus *Prevotellaceae-NK3B31 group* (*P* < 0.05) respectively. These results provide us with new insights into the cause of the gut microbiome and stress, and the contributions of gut microbiome in metabolic and physiological regulation in response to weaning stress.

## Introduction

Gut microbiota is known to play an essential role in host physiological regulation, including digestion, metabolism and immune. However, the gut microbial ecology can be disturbed by stress, such as diet modulation, circadian rhythms shifting, and even psychological pressure. For instance, regrouping social stress reduced gut microbial diversity in Syrian hamsters (Partrick et al., 2018). Mental stress decreased the gut microbial richness and diversity in rodents (Winter et al., 2018). Six weeks of restraint stress decreased the gut proportion of *Lactobacillus* in mice (Dodiya et al., 2020). Weaning is one of the most stressful events in pig life involving psychological, physiological, nutritional, and cognitive-behavioral responses (Campbell et al., 2013). Reduction of feed intake and body weight gain, increase in plasma cortisol level, and high diarrhea frequency often emerges in weaned piglets. During suckling period, piglets have built up a milk-oriented microbiota. While at weaning day, piglets are abruptly separated from the sows and transported to a different physical environment, and regrouped with piglets from other litters, changed from suckling sow milk to feeding on solid feed. Thus, weaning piglets is a typical model for understanding the stress effects on the gut microbiome and the overall health. Researches indicated that the cecal and fecal microbial communities were different before and after weaning (Frese et al., 2015). The colonic microbial composition and functions of piglets on the third day of weaning were different from that of their suckling period (Li et al., 2018). However, the dynamic composition and diversity of gut microbiome shift over time (Grosicki et al., 2018). The different gut microbiome before and after weaning can not exclude the developmental effects. Therefore, comparison of the dynamics of gut microbiota between the suckling and weaned piglets paired in litters is of interest to elucidate the effects of weaning stress.

Researches indicated that the role of metabolites in mediating the interaction of gut microbiome and host physiology. For example, short-chain fatty acids (SCFAs), including acetic, propionic, and butyric acid, which are the primary bacterial metabolites in the gut, act as signal molecules inhibit histone deacetylation, play positive actions on the homeostasis of carbohydrate and lipid metabolism, gastrointestinal motility and immunity by directly binding to their specific G-protein-coupled receptor (GPR) 41 and GPR43 (Wong et al., 2006; Koh et al., 2016). Rectal administration of butyric acid improved the intestinal motility, reduced the abdominal pain or discomfort (Koh et al., 2016). Gutierrez et al. (2020) observed that metabolites carbohydrates, sugar alcohols and bile acids, and carboxylic acids and secondary bile acids also could influence *Candida albicans* colonization in the gastrointestinal tract (Gutierrez et al., 2020). Therefore, for understanding the interaction between host physiology and the colonic microbiome under weaning stress, this study was designed to investigate the effects of weaning stress on the colonic microbiome in piglets, and the relationships between microbiome and metabolites, and the salivary cortisol concentration.

## Materials and Methods

### Animals and Sampling

All animal care and experimental protocols were following the Guidelines of the Institutional Animal Care and Use Committee of the Institute of Animal Husbandry and Veterinary Sciences, Shanghai Academy of Agricultural Sciences, and complied with standard international regulations.

According to the principle of full compatriot pairing, 14 Meishan female piglets (from 3 l with same parity) paired in litter and body weight (4.51 ± 0.22 kg) were allocated into the suckling group and the weaning group, eight piglets in each group. The piglets in the weaning group were weaned at 21 days old and were free access to a typical nursery diet based on corn and soybean meal, while the suckling group continued to stay with sows. The diet was formulated to meet the estimates of nutrient requirements of weaned piglets. In addition to separation from sows and different dietary sources, the weaning piglets kept in the same environment with suckling group in this experiment. Throughout the 48 h experiment, all piglets were free access to water. The bodyweights of piglets were recorded before and after the experiment.

After 48 h of experiment, saliva was collected using cotton swab (Thomsson et al., 2014), in brief, held the cotton swab with forceps and allowed the piglet to chew on the cotton swab until it was soaked. Then the saliva was recovered by centrifuging the cotton swab at 3000 × *g* in room temperature for 5 min (GTR10-1 centrifuge, Era Beili Centrifuge, Co., Ltd., Beijing, China) and stored at −20°C for determination of cortisol content. After collecting saliva, we euthanized piglets with CO_2_ gas followed by sample (Petrosus et al., 2018). The colonic digesta was collected and stored on ice for transportation to the lab where each sample was divided into three parts for different analysis. One part (0.3 g of digesta) was kept in tubes that contained 0.9 mL ethanol, gently mixed with ethanol and then stored at −20°C until DNA extraction, another part (2.0 g of digesta) was immediately kept frozen at −80°C until metabolome analysis, and the third part (1.0 g of digesta) for SCFAs analysis was kept frozen at −20°C.

### Determination of Salivary Cortisol Concentration

As compared to blood sampling, salivary sample collection is non-invasive with less interference from acute stress, and salivary steroid hormones can reflect the biological activities of steroid hormones in plasma (Thomsson et al., 2014). Thus for avoiding blood sampling stress, here we measured the salivary cortisol concentration using the commercial EIA kits (Cayman, Cortisol, Item No. 500360, Intra-assay CV < 5.1%, Inter-assay CV < 6.7%, sensitivity is 110 pg/mL). All samples were run in triplicate.

### Pyrosequencing Analysis of Microbiome in the Colonic Digesta

The total genomic DNA of colonic bacteria was extracted from the colonic digesta according to the manufacturer’s instruction of the commercial DNA extraction kit (Ultra clean fecal DNA isolation kit, Solarbio, Co., Ltd., China). And the concentration of the extracted DNA was measured via Nano-Drop 1000 spectrophotometer (Thermo Scientific, Inc., Wilmington, DE, United States) at wavelengths of 260 and 280 nm. The integrity of the DNA extracts was assessed by electrophoresis on 1.0% agarose gels. For the 16S rRNA gene sequencing, the primers 515F (5′-GTGCCAGCMGCCGCGG-3′) and 907R (5′-CCGTCAATTCMTTTRAGTTT-3′) were used to amplify the V4 through V5 hypervariable regions of the 16S rRNA gene. The PCR reaction pool contains 4 μL 5 × FastPfu Buffer, 2 μL dNTPs (2.5 mM), 0.4 μL FastPfu Polymerase, 0.8 μL of each primer (5 μM), and 10 ng of DNA as the template. The following PCR cycles were used: initial denaturation at 95°C for 5 min, 27 cycles at 95°C for 30 s, 55°C for 30 s, and 72°C for 45 s, and a final extension at 72°C for 10 min. The amplification products from each sample were evaluated by electrophoresis in 2% agarose gels and purified using the QIAquick PCR purification kit (Qiagen, Valencia, CA, United States). The products were quantified using QuantiFluor™-ST fluorescent quantitation system (Promega, Madison, WI, United States), and then mixed in equivalent proportions. Sequencing was performed using Illumina Miseq PE250 according to the manufacturer’s instructions.

The 16S rRNA gene sequences were processed using the Mothur software to remove low-quality sequences (Schloss et al., 2009). The operational taxonomic units (OTU) picking with 97% similarity cut off was compiled with Qiime using default parameters. Taxonomic classification was performed based on the OTU database. The microbial alpha-diversity indices (including Chao, Ace, Shannon and Simpson) were calculated with Mothur program^1^ and Rarefactuin software. The community difference between the suckling and weaning group was evaluated using principal co-ordinates analysis (PCoA) and Adonis test, and a significant difference was assigned at *P* < 0.05.

### Verification of Differential Bacteria by Quantitative Real-Time PCR (qRT-PCR)

To quantify *Lactobacillus* and *Prevotellaceae*, different primers were used: F-lac 5′-AGCAGTAGGGAATCTTCCA-3′ and R-lac 5′-CACCGCTACACATGGAG-3′ for *Lactobacillus*; F-pre 5′-AACCCGTTGGGTGTGCC-3′ and R-pre 5′-AGIGCCCAAACCTCCATCTCTCC-3′ for *Prevotellaceae*. The primers were synthesized commercially by Sangon Biotech (Shanghai, China). The DNA of each sample was mixed equally as a template for PCR amplification. PCR mixture (50 μL) contained 25 μL Es Taq MaserMix (CWBIO, Beijing, China), 40 ng bacterial template DNA, 0.4 μM primer. The PCR program consisted of 35 cycles with a DNA denaturation step at 95°C (30 s), followed by an annealing step at 60°C (30 s) and elongation step at 72°C (30 s). The PCR was completed with a final elongation step at 72°C (2 min). The PCR products were purified with the commercial DNA purification kit (Solarbio, Beijing, China), and the concentrations were measured. Products obtained were also sequenced for confirmation (Sangon Biotech, Shanghai, China), and calculated the number of copies. Serial dilutions were performed, and 10^–2^ ∼ 10^–9^ copies of the gene per reaction were used for calibration. The functions describing the relationship between C_*t*_ (threshold cycle) and x (log copy number) for the different assays were: C_*t*_ = −3.38x + 32.20, R^2^ = 0.99 for *Lactobacillus*; C_*t*_ = −3.27x + 36.42, *R*^2^ = 0.99 for *Prevotellaceae*.

The qRT-PCR was performed with the QuantStudio5 Real-Time PCR Systems (ABI, United States). The PCR reaction pool contained 10 μL SYBR Green buffer (Takara, Japan), 0.4 μL ROXII, 0.8 μL of each primer (10 μM) and 40 ng of DNA samples. The reaction condition was 95°C for 1 min, 35 cycles of 95°C for 15 s, 60°C for 30 s and 72°C for 30 s. And then, the product melting curve was formulated to determine the specificity of amplification.

### Determination of SCFAs

For the SCFAs detection, 1.0 g of each colonic digesta sample was diluted with distilled water, homogenized, and centrifuged (Heraeus Instruments, Düsseldorf, Germany) at 11,900 × *g* for 15 min. The liquid supernatant was filtrated through a 0.22 μm filter, then 0.5 μL filtrate was injected into the gas chromatograph (7890B, Agilent Technologies, Santa Clara, CA, United States) equipped with a capillary column (30 m × 0.32 mm × 0.25 μm film thickness; Varian, Inc., United States). To measure the SCFAs, 1.5 mM crotonic acid was used as an internal standard. The column, injector and detector temperatures were 130, 180, and 180°C, respectively. A standard SCFAs mixture containing acetic acid, propionic acid, butyric acid, pentanoic acid and isopentanoic acid were used for calculation, and the results were expressed as mg/g of a fresh digesta sample.

### Gas Chromatograph-Mass Spectrometer (GC–MS) Analysis of the Metabolite Profiles in the Colonic Digesta

The colonic digesta (50 mg) were mixed with 360 μL of cold methanol and 40 μL of internal standard (0.3 mg/mL, L-2-chloro-phenylalanine). All samples were ground to a fine powder using Grinding Mill at 65 Hz for 90 s. The samples after ground were added 200 μL of chloroform and 400 μL of distilled water, vortex-mixed for 1 min and ultrasonically extracted in an ice water bath for 30 min. After centrifuged at 10000 rpm and 4°C for 10 min, 400 μL of supernatant was transferred to a vial for GC–MS analysis.

Each 1 μL aliquot of the derived sample was injected into a 7890A-5975C GC system (Agilent Technologies, Santa Clara, CA, United States) equipped with HP-5MS capillary column (30 m × 0.25 mm × 0.25 μm, Agilent J&W Scientific, Folsom, CA, United States). The inlet temperature was set to 260°C, and Helium was used as the carrier gas at a constant flow rate of 1.0 mL/min through the capillary column. The column temperature was initially set at 60°C and last for 2 min, and then increased at a rate of 8°C/min to 310°C and held for 6 min.

The original data of GC–MS is preprocessed by ChromaTOF software (v4.34, LECO, St. Joseph, MI, United States) to derive a three-dimensional data matrix in CSV format. The positive and negative data were combined to get a combined data set that was imported into SIMCA-P + 14.0 software package (Umetrics, Umeå, Sweden). Principal component analysis (PCA) was carried out to visualize the metabolic alterations between the suckling group and weaning group. The metabolites with variable importance in the projection (VIP) values of 1.0 and *P*-values of 0.05 (threshold) were selected as metabolites that could discriminate between the suckling group and weaning group. The effects of weaning on the metabolic pathways and metabolite set enrichment analysis were evaluated based on a tool for metabolomic data analysis, which is available online^2^ (Xia et al., 2009).

### Statistical Analysis

The data were analyzed with SPSS 17.0 using a randomized block design, considering the weaning as the main effect and the replicate as a block. The effects of weaning on the salivary cortisol content, microbiome and metabolites in the colon of the piglets were tested for significance using the Paired-samples *t*-test, with significant differences defined as *P* < 0.05. Results were presented as means value ± standard error of the means (SEM). The correlations between the metabolites and bacterial compositions in colonic contents and the salivary cortisol levels were assessed by Pearson’s correlation test using R language and Pheatmap package.

## Results

### Bodyweight and Salivary Cortisol Level

**FIGURE 1:**
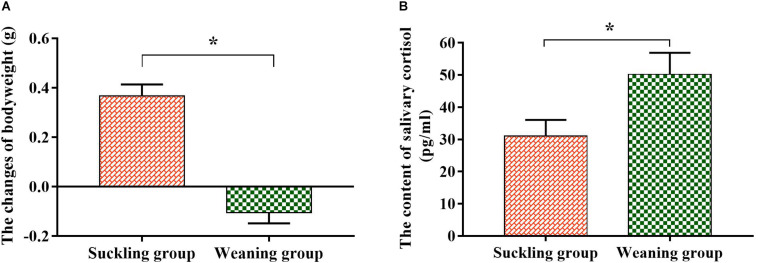
The changes of the bodyweight and salivary cortisol content of piglets. **(A)** Bodyweight change; **(B)** salivary cortisol content. Graph shows means value ± SEM (*N* = 7). *(*P* < 0.05) means significant difference between the suckling group (orange column) and the weaning group (green column).

After 48 h of experiment, the bodyweight of the suckling piglets increased by 0.37 ± 0.04 g, while the weaned piglets decreased by 0.11 ± 0.04 g, and there was a significant difference between the two groups (*P* < 0.01, Figure 1A). The EIA assay illustrated that the salivary cortisol concentration of the weaned piglets increased significantly (*P* = 0.04, Figure 1B) compared to their suckling littermates, which also indicated that weaned piglets were in stress state at this time.

### Colonic Microbiota

**FIGURE 2:**
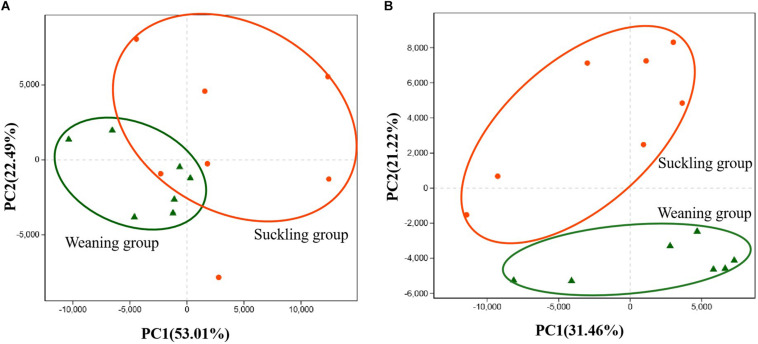
Principal co-ordinates analysis (PCoA) plots. **(A)** PCoA on phylum level; **(B)** PCoA on genus level. Adonis test showed weaning has a significant impact on the colonic microbial community (*R*^2^ = 0.22, *P* = 0.02 at the phylum level) and (*R*^2^ = 0.21, *P* = 0.01 at the genus level). Orange dots represent the suckling piglets, green triangles represent the weaned piglets, *N* = 7.

Pyrosequencing profiled a total of 1,064,854 valid reads that were assigned to 991 OTUs after screening them with strict criteria. As shown in Table 1, at a genetic distance of 3%, no differences were found in the number of OTUs, coverage, diversity indices (Shannon and Simpson) and richness estimators (Ace and Chao) of the colonic microbiota between the two groups. PCoA visually confirmed the distinct separation of microbial communities at the phylum level (*P* = 0.04) and genus level (*P* = 0.02) between the suckling and weaned piglets (Figure 2).

**TABLE 1:** Diversity estimation of the 16S rRNA gene libraries from microbiota in the colon of piglets.

Item	Suckling group	Weaning group	*P*-value
OTUs	942 ± 14.02	920 ± 22.29	0.39
Coverage	0.99 ± 0.00	0.99 ± 0.00	0.60
Ace	531.36 ± 68.84	546.14 ± 72.00	0.88
Chao	533.69 ± 70.80	550.11 ± 68.84	0.87
Shannon	4.06 ± 0.26	4.31 ± 0.23	0.49
Simpson	0.05 ± 0.00	0.03 ± 0.00	0.28

*The operational taxonomic units (OTUs) were defined with 97% similarity. The coverage percentages, richness estimators (Ace and Chao), and diversity indices (Shannon and Simpson) were calculated. The data were expressed as means value ± SEM, *N* = 7. *P* < 0.05 means significant difference between the suckling group and the weaning group.*

**FIGURE 3:**
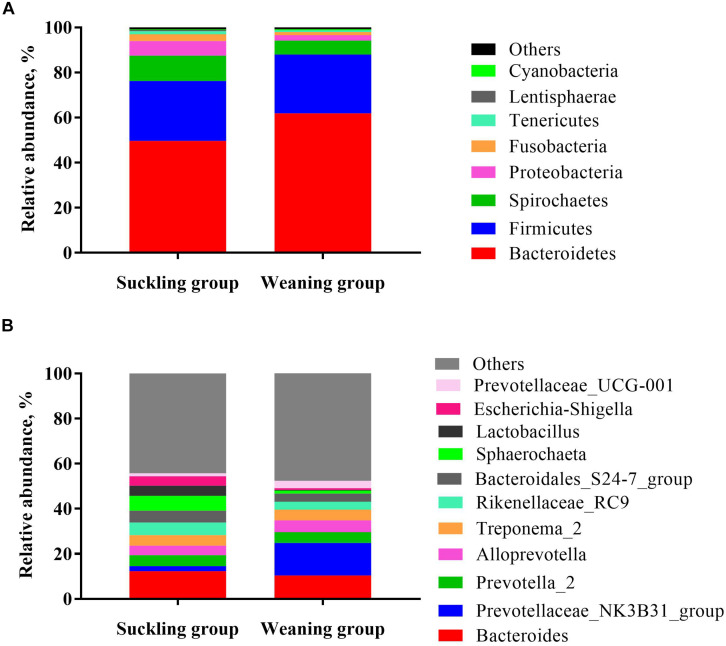
Taxonomic classification of the 16S rRNA gene sequences at the **(A)** phylum and **(B)** genus levels for the suckling and weaning group.

**FIGURE 4:**
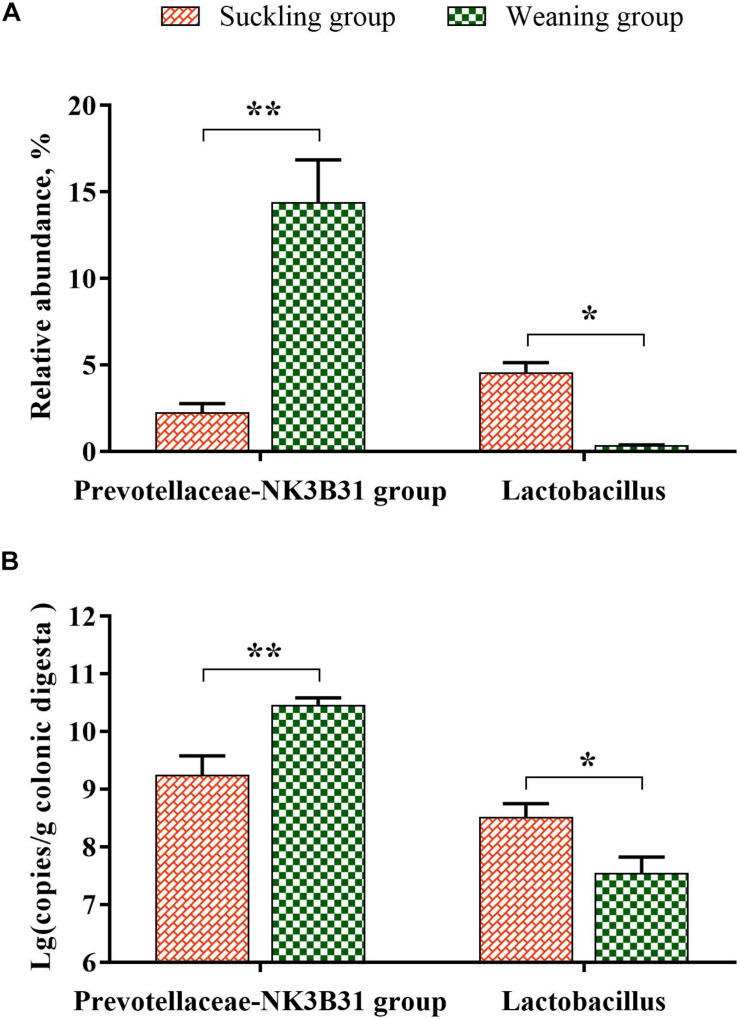
The column chart identifying the significantly different taxa between the suckling group and the weaning group at the genus level. **(A)** The results of pyrosequencing; **(B)** the verification results of qPCR (log 16S rRNA gene copy number/g fresh matter). Graph shows means value ± SEM (*N* = 7), * significant difference (*P* < 0.05) and ** significant difference (*P* < 0.01) between the suckling group (orange column) and the weaning group (green column).

At the phylum level (Figure 3A), the bacterial sequences were composed predominantly of the phylum *Bacteroidetes* (55.78%), *Firmicutes* (26.36%), *Spirochaetes* (8.92%), *Proteobacteria* (4.46%), *Fusobacteria* (2.21%), *Tenericutes* (1.02%) and 12 other phyla that collectively comprised 1.25% of the total sequences analyzed. Moreover the relative abundance of *Bacteroidetes* was significantly higher in the weaning group (*P* = 0.02), but the relative abundance of *Lentisphaerae* was significantly lower in the weaning group than that in the suckling group (*P* = 0.03). At the genus level (Figure 3B), *Bacteroides* was the top enriched in the suckling group, while *Prevotellaceae-NK3B31 group* was the most abundant genera in the colonic microbial community of the weaned piglets. Of the top 15 genera, the relative abundance of *Lactobacillus* was decreased significantly (*P* < 0.01), and the abundance of *Prevotellaceae-NK3B31 group* was increased significantly (*P* = 0.02) in the colonic microbiota of weaned piglets (Figures 3B, 4A). Meanwhile, qRT-PCR obtained a consistent result (Figure 4B).

### Correlation Analysis Between Cortisol Level and Colonic Differential Microbe

In order to evaluate the associations between the salivary cortisol level and the colonic differential microbe, Pearson’s correlation coefficient was calculated (Table 2). The salivary cortisol level was significantly positively associated with the relative abundance of phylum *Bacteroidetes* (*r* = 0.81, *P* = 0.01), and genus *Prevotellaceae-NK3B31 group* (*r* = 0.76, *P* = 0.03). However, no statistical correlation was observed between the salivary cortisol level and the proportion of phylum *Lentisphaerae* (*r* = −0.54, *P* = 0.45), and the genus *Lactobacillus* (*r* = −0.35, *P* = 0.06).

**TABLE 2:** Pearson correlation analysis between cortisol level and differential microbe.

	Taxonomy	Correlation	*P*-value
Phylum	*Bacteroidetes*	0.81	0.01
	*Lentisphaerae*	–0.54	0.45
Genus	*Prevotellaceae -NK3B31*	0.76	0.03
	*Lactobacillus*	–0.35	0.06

**P* < 0.05 indicates significant correlation between cortisol level and differential microbe.*

### Metabolomic Profiles

**FIGURE 5:**
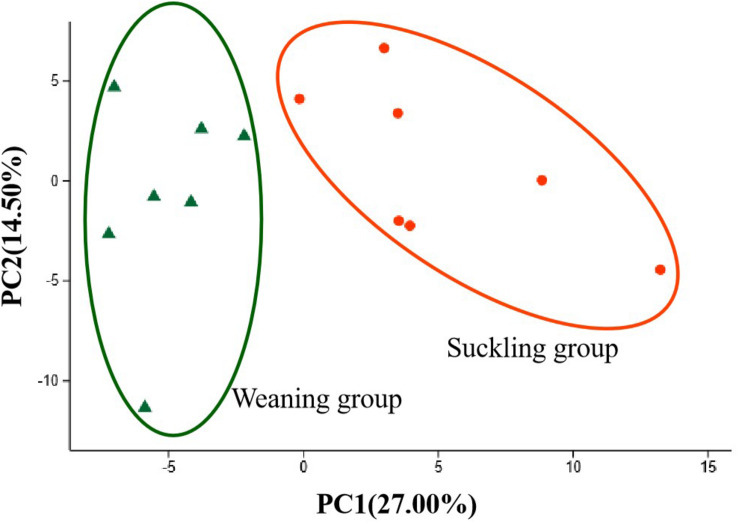
PCA score plots of metabolies in colonic digesta of piglets between the suckling group and the weaning group. Orange dots represent the suckling piglets, green triangles represent the weaned piglets, *N* = 7.

The typical total ion-current chromatogram showed several 100 peaks in a single analysis, and after the deconvolution of the chromatograms, the quantitative and qualitative information was obtained. In general, a total of 169 non-targeted peaks/metabolites were detected. PCA results showed that the variation in the microbial metabolites could be differentiated readily according to the different groups (Figure 5). To identify which metabolites were responsible for this difference, the parameters of VIP > 1 and *P* < 0.05 were used as criteria. Accordingly, 18 metabolites in the colonic contents were annotated and listed in Table 3. Compared to the suckling piglets, the weaned piglets had significant higher concentrations of arabitol (*P* < 0.01), benzoic acid (*P* < 0.01), caprylic acid (*P* < 0.01), cholesterol (*P* = 0.01), digitoxose (*P* = 0.01), dihydrocholesterol (*P* = 0.04), galactinol (*P* = 0.01), glucose (*P* < 0.01), glycerol (*P* < 0.01), kynurenine (*P* = 0.02), oxamic acid (*P* = 0.01), phenol (*P* < 0.01), phenylacetic acid (*P* = 0.01), propane-1,3-diol (*P* = 0.01) in the colonic digesta, but significantly lower levels of citric acid (*P* = 0.03), hexadecylglycerol (*P* = 0.02), maltotriose (*P* = 0.03), and myo-inositol metaboltes (*P* = 0.03).

**TABLE 3:** The colonic metabolites that differed between suckling group and weaning group.

Pathway	Metabolite	VIP^*a*^	FC^*b*^	*P*-value
Amino acid	Benzoic acid	1.16	0.32	0.00
	Phenol	1.92	0.65	0.00
	Phenylacetic acid	1.08	0.54	0.01
Carbohydrate	Arabitol	1.87	0.15	0.00
	Citric acid	1.40	5.27	0.03
	Galactinol	1.09	0.32	0.01
	Glucose	1.38	0.20	0.00
	Glycerol	4.43	0.24	0.00
Lipid	Caprylic acid	1.10	0.71	0.00
	Cholesterol	1.29	0.52	0.01
	Dihydrocholesterol	1.33	0.47	0.04
	Hexadecylglycerol	2.11	27.38	0.02
	Glycerol	4.43	0.24	0.00
	Propane-1,3-diol	1.00	0.36	0.01
Nucleotide	Oxamic acid	1.59	0.57	0.01
Others	Digitoxose	1.23	0.19	0.01
	Kynurenine	1.97	0.15	0.02
	Maltotriose	3.24	29.50	0.03
	Myo-inositol	5.60	17.17	0.03

*^*a*^VIP, variable importance in the projection. ^*b*^FC, fold change, means value of peak area obtained from the suckling group/mean value of peak area obtained from the weaning group. If the FC value less than 1, it means that metabolites are less in the suckling group than in the weaning group.*

Meanwhile, the gas chromatograph analysis profile of SCFAs illustrated that the weaned piglets had significantly higher acetic acid (*P* = 0.02, Table 4), propionic acid (*P* = 0.01) and total SCFAs concentrations (*P* = 0.01) in the colonic digesta than that of the suckling piglets. However, there were no differences in the concentrations of butyric acid (*P* = 0.07), pentanoic acid (*P* = 0.15), and isopentanoic acid (*P* = 0.06) in the colonic digesta between the weaned and suckling piglets.

**TABLE 4:** Short chain fatty acids (SCFAs) concentrations (mg/g) in the colon of piglets.

Item	Suckling group	Weaning group	*P*-value
Acetic acid	2.69 ± 0.34	4.13 ± 0.44	0.02
Propionic acid	0.81 ± 0.15	1.53 ± 0.17	0.01
Butyric acid	0.21 ± 0.06	0.40 ± 0.07	0.07
Pentanoic acid	0.21 ± 0.04	0.12 ± 0.04	0.15
Isopentanoic acid	0.10 ± 0.02	0.04 ± 0.02	0.06
Total SCFAs	4.02 ± 0.48	6.23 ± 0.66	0.01

*The data were expressed as means value ± SEM, *N* = 7. ^∗^*P* < 0.05 means significant difference between the suckling group and the weaning group.*

**FIGURE 6:**
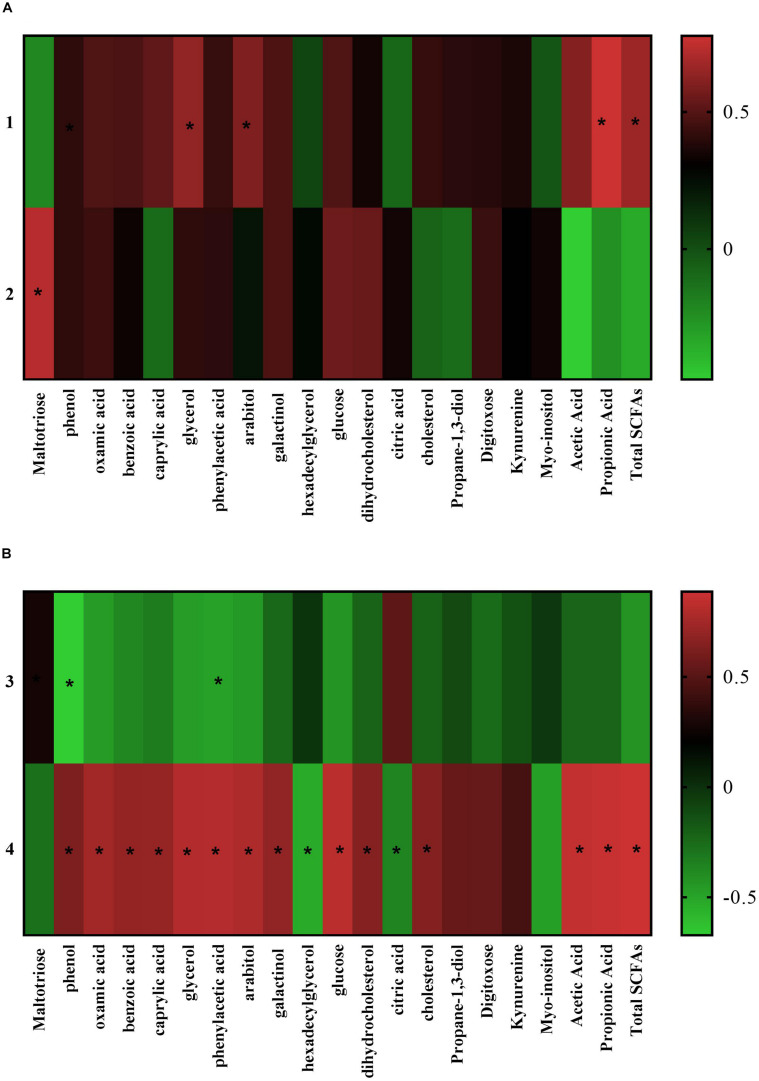
Pearson correlation analysis between the different colonic microbiota and different metabolites (only metabolites with significant correlation are listed). **(A)** At the phylum level; **(B)** at the genus level. (1) *Bacteroidetes*; (2) *Lentisphaerae*; (3) *Lactobacillus*; (4) *Prevotellaceae_NK3B31_group*. The cells are colored based on the Pearson correlation coefficient between the different bacteria (relative abundance) and metabolites (VIP > 1.0 and *P* < 0.05) in the colon, red represents positive correlation, green represents negative correlation, and * represents that the correlation was significant (*P* < 0.05).

The Pearson’s correlation analysis between the colonic microbiota at the phylum level and metabolome (Figure 6A) revealed the proportion of *Bacteroidetes* in the colonic microbiome was positively related to the concentration of glycerol (*r* = 0.55, *P* = 0.04), arabitol (*r* = 0.48, *P* = 0.05), propionic acid (*r* = 0.78, *P* = 0.00), and total SCFAs (*r* = 0.58, *P* = 0.05). While the abundance of phylum *Lentisphaerae* was positively related to maltotriose (*r* = 0.69, *P* = 0.01). At the genus level (Figure 6B) the abundance of genus *Lactobacillus* in the colonic microbiome was negatively related to the concentration of phenylacetic acid (*r* = −0.53, *P* = 0.04) and phenol (*r* = −0.67, *P* = 0.01) in the colonic digesta. While the abundance of genus *Prevotellaceae-NK3B31 group* positively associated with the level of benzoic acid (*r* = 0.64, *P* = 0.01), phenol (*r* = 0.54, *P* = 0.04); phenylacetic acid (*r* = 0.78, *P* = 0.00), and carbohydrate metabolites (arabitol, *r* = 0.75, *P* = 0.00; galactinol, *r* = 0.62, *P* = 0.02; glucose, *r* = 0.80, *P* = 0.00), and nucleotide metabolites (oxamic acid, *r* = 0.70, *P* = 0.01), and lipid metabolites (caprylic acid, *r* = 0.64, *P* = 0.01; cholesterol, *r* = 0.56, *P* = 0.04; glycerol, *r* = 0.77, *P* = 0.00; dihydrocholesterol, *r* = 0.57, *P* = 0.03), and SCFAs (acetic acid, *r* = 0.84, *P* = 0.00; propionic acid, *r* = 0.86, *P* = 0.00; and total SCFAs, *r* = 0.89, *P* = 0.00). Meanwhile, the proportion of *Prevotellaceae-NK3B31 group* negatively associated to metabolites: citric acid (*r* = −0.44, *P* = 0.01) and hexadecylglycerol (*r* = −0.56, *P* = 0.04) of the colonic digesta.

## Discussion

The hypothalamic–pituitary–adrenal (HPA) axis is a stress-responsive neuroendocrine system, in which the glucocorticoid hormones corticosterone in rodents and cortisol in other mammals act as the end-products of HPA. In mammals, the physiological stress is typically characterized by the increased level of glucocorticoids in blood and saliva (Sapolsky et al., 2000; Jarvis et al., 2006; Rutherford et al., 2006). For instance, 3 weeks of feed restriction increased the plasma cortisol level in growing pigs (Metges et al., 2015). Weaning stress piglets had increasing plasma cortisol concentrations and growth reduction (Moeser et al., 2007; He et al., 2011; Tuchscherer et al., 2018). The acute elevation of glucocorticoids elicits energy metabolism from various tissues and supply enough glucose into the circulation to maintain homeostasis and copy with the fight or flight response (Kanitz et al., 2012; Turan et al., 2015). Furthermore the increased cortisol level may induce peripheral insulin resistance, impair the hypothalamic function of appetite regulation, and consequently reduced in feed intake and body weight gain (Geer et al., 2014). Present data agree with above, the increase in salivary cortisol level and reduction of body weight in the weaned piglets compared to their suckling litter mates indicate that the weaned piglets were in stress.

Accompanied by the weaning stress, gut microbial diversity and composition shifted (Chen et al., 2017; Li et al., 2018). Here the colonic bacterial communities of the weaned piglets were distinctly separated from their suckling littermates at genus level in PCA, and the weaned piglets had a higher proportion of phylum *Bacteroidetes* than their suckling littermates. Phylum *Bacteroidetes* have a superb ability to utilize the nutrients including simple and complex sugars and polysaccharides for its growth, so as can adapt to environmental changes and stresses (Wexler, 2007). Increased phylum *Bacteroidetes* also were observed in post-traumatic stressed mice (Gautam et al., 2018), heat stressed birds (Shi et al., 2019; Zhu et al., 2019). Consistently, here the phylum *Bacteroidetes* was positively associated with the concentration of glycerol and arabitol in the colonic digesta, which are metabolites in the carbohydrate metabolic pathway.

Opposite to the increased level of *Bacteroidete* phylum in the colonic microbiota, the proportion of *Lentisphaerae* phylum decreased in the weaned piglets. Researches observed that *Lentisphaerae* phylum was associated with improved health in human (Jiang et al., 2015), increased feed utilization and growth performance in cattle (Myer et al., 2015) and pigs (McCormack et al., 2019). Decreased *Lentisphaerae* phylum was observed in post-traumatic stress disorder patients (Hemmings et al., 2017). These suggest that here the reduction of *Lentisphaerae* phylum in the colonic microbial community may relate to the bodyweight reduction in the weaned piglets. Whereas, the potential role of *Lentisphaerae* phylum in the gut should be elucidated in further study.

Interestingly, *Bacteroides* was the first predominant genus in the colonic microbiota of the suckling piglets, while *Prevotellaceae-NK3B31 group* was the first predominant genus in the colon of the weaned piglets. During the weaning transition, the fecal microbial community of piglets had a distinct shift from *Bacteroides* to *Prevotella* as the most abundant genus (Alain et al., 2014). Frese et al. (2015) observed in piglets that the relative abundances of *Bacteroidaceae* in the fecal microbiota declined from birth to 7 weeks of age, while that of *Prevotellaceae* increased (Frese et al., 2015). Moreover, in the piglet gut microbiome, *Prevotella* was present in nursing piglets with a relatively low abundance and increased in weaned piglets when a plant-based diet was introduced (Guevarra et al., 2018). Researches indicated that the proportion of *Bacteroides* in the gut was associated with the consumption of animal protein and fat (Wu et al., 2011). Whereas, *Prevotellaceae* is one of the predominant fiber-degrading bacterial species in the intestinal tracts of pigs (Ivarsson et al., 2014). The *Bacteroides*-driven enterotype is predominant with a high intake of protein and animal fat, whereas the *Prevotella*-driven enterotype appears predominant in individuals that consume diets rich in carbohydrate and fiber (Wu et al., 2011; de Moraes et al., 2017). Consistently, the abrupt change in diet from milk to plant-based solid feed caused an increase in the abundance of the genus *Prevotellaceae-NK3B31 group* and a high level of acetic acid, propionic acid in the colonic digesta. The proportion of the genus *Prevotellaceae-NK3B31 group* in the colonic bacterial community was positively associated with the concentration of acetic acid, propionic acid and total SCFAs in the colonic digesta. SCFAs are carbohydrate metabolites, and the acetic acid and propionic acid were the most abundant SCFAs in the colonic content. Acetic and propionic acids may act by binding to the GPR41 and GPR43, which express in the colon, liver, adipose and skeletal muscle, regulate the secretions of glucagon-like peptide-1 (GLP-1) and peptide YY (PYY), attenuate insulin resistance, and thereby affect systemic lipid and glucose metabolism. Observation in healthy men indicated that intravenous infusion of sodium acetate inhibited lipolysis, and reduced the plasma glycerol and free fatty acid concentrations (Suokas et al., 1988). Studies in mice also illustrated that the treatments of acetate and propionate increased hepatic glycogen synthesis (Weitkunat et al., 2016). Here the genus *Prevotellaceae-NK3B31 group* positively correlated with the colonic metabolites caprylic acid, glycerol, cholesterol, dihydrocholesterol, phenylacetic acid, phenol, galactinol, and oxamic acid, which were in the lipid, carbohydrate, amino acid, and nucleotide metabolic pathway. On the contrary, suckling piglets had a higher level of citric acid and hexadecylglycerol in the colonic digesta compared to the weaned piglets, and both metabolites negatively correlated with the proportion of the *Prevotellaceae-NK3B31 group* in the colonic microbial community. Citric acid, known as tricarboxylic acid (TCA) cycle, is involved in glucose and energy synthesis (Nagai et al., 2010). Hexadecylglycerol acts as the ether lipid precursor in the lipid metabolic pathway (Bergan et al., 2014). Thus, the predominant genus shifting from *Bacteroides* to *Prevotellaceae-NK3B31 group* in the colon may indicate a metabolism change, including amino acid, and carbohydrate and lipid, and nucleotide.

Recently, there is an increasing concern about the relationship between stress and gut microbiota. Dexamethasone in drinking water can inhibit the colonization of probiotics in the gut, and increase the risk of invasion by pathogenic intestinal bacteria (Yang et al., 2016). The serum cortisol level increased during the weaning period when the piglets often infected with pathogenic bacteria (Moeser et al., 2007; Tuchscherer et al., 2018). The population of *Streptococcus* (a zoonotic pathogen) in the gut was increased in weaned piglets (Su et al., 2008). Mudd et al. (2017) observed in piglets that the fecal *Ruminococcus* population negatively correlated to the serum cortisol level (Mudd et al., 2017). Here, the salivary cortisol level positively associated with the relative abundance of *Bacteroidetes* phylum, and *Prevotellaceae-NK3B31* genus. *Bacteroidales* and *Prevotellaceae* are Gram-negative bacteria (Goncalves Pessoa et al., 2019), which can produce lipopolysaccharide (LPS, bacterial endotoxin). LPS may cause an acute inflammatory response, stimulate the release of cortisol (Wright et al., 2000; Singh et al., 2018), tumor necrosis factor-alpha (TNFα), interleukin-1β (IL-1β), gamma interferon (IFNγ) and other inflammatory cytokines and chemokines (Everhardt Queen et al., 2016; Shin and Ajuwon, 2018). Depression patients had an increased plasma cortisol level and proportion of *Bacteroides* (Kelly et al., 2015) and *Prevotella* genus in the gut microbiome (Lin et al., 2017). Therefore, weaning caused the increase of the relative abundance of *Bacteroides* and *Prevotella* in the colonic microbial community may induce high production of LPS, which can stimulate the cortisol secretion.

Genus *Lactobacillus* can produce lactic acid and has benefits for health in human and other animals (Kleerebezem et al., 2003). The high population of *Lactobacillus* in the gut can inhibit the growth of pathogenic bacteria (Pfeiler and Klaenhammer, 2007; Valeriano et al., 2014), reduce inflammatory response triggered by LPS (Griet et al., 2014). Administration of *Lactobacillus* can prevent stress-induced changes in neurogenesis, barrier integrity, and stress reactivity (Logan and Katzman, 2005; Messaoudi et al., 2011; Ait-Belgnaoui et al., 2012; Aizawa et al., 2016). Liang et al. (2015) observed that *Lactobacillus Helveticus NS8* down-regulated the secretion of corticosterone in Sprague Dawley rat (Liang et al., 2015). Conversely, reduction of intestine *Lactobacillus* counts was associated with various stress, such as no bedding, food, or water (Tannock and Savage, 1974), and maternal separation (Bailey and Coe, 1999). Zijlmans et al. (2015) also observed that pregnant women with high self-reported stress had a high level of cortisol in saliva and low abundances of *Lactobacillus* in the fecal microbiome (Zijlmans et al., 2015). Administration of dexamethasone decreased the population of *Lactobacillaceae* in the colonic microbiome (Huang et al., 2015). Present data are in line with above, and the salivary cortisol level had a negative association tendency with the proportion of *Lactobacillus* in the colonic microbiota, moreover, the abundance of *Lactobacillus* genus negatively correlated with the concentrations of phenylacetic acid and phenol in the colonic digesta, these two metabolites are in the amino acid metabolic pathway. Addition of *Lactobacillus* in feed improved the apparent protein digestibility in weaned piglets (Dowarah et al., 2018; Wang et al., 2019). These suggests that the decrease in the proportion of *Lactobacillus* in the colonic microbial community may result in a reduction of protein digestibility, and this may account for the decrease of body weight in weaned piglets. Therefore, considering the interaction of *Lactobacillus* and stress, and the advantage of *Lactobacillus* for health and feed utilization, the addition of *Lactobacillus* for piglet can be a practical strategy for weaning stress amelioration.

## Conclusion

The present work demonstrated that weaning caused an increase of salivary cortisol level, alteration in the colonic microbiota and metabolome profiles. The salivary cortisol level positively associated with the colonic *Bacteroidetes* phylum and *Prevotellaceae-NK3B31 group* genus and negatively associated with the phylum *Lentisphaerae.* The colonic *Bacteroidetes* phylum associated with carbohydrate metabolic pathway, *Lactobacillus* genus associated with the amino acid metabolic pathway, and the *Prevotellaceae-NK3B31 group* genus related to the amino acid, carbohydrate, lipid, and nucleotide metabolic pathways. These results provide us with new insights into the cause of the gut microbiome and stress, and the contributions of the gut microbiome in metabolic and physiological regulation in response to weaning stress.

## Data Availability Statement

The datasets presented in this study can be found in online repositories. The names of the repository/repositories and accession number(s) can be found at: https://www.ncbi.nlm.nih.gov/genbank/, with accession number AP018405.1; and at https://www.ncbi.nlm.nih.gov/genbank/, with accession number AP014597.1.

## Ethics Statement

The animal study was reviewed and approved by the Guidelines of the Institutional Animal Care and Use Committee of the Institute of Animal Husbandry and Veterinary Sciences, Shanghai Academy of Agricultural Sciences.

## Author Contributions

DX: conception and design. XJ, NL, HZ, and HY: animal feeding, sampling, and determination. XJ: data analysis and drafting the manuscript. HL: diet formulation. XJ, NL, HZ, HY, DX, and HL: final approval of the manuscript. All authors contributed to the article and approved the submitted version.

## Conflict of Interest

The authors declare that the research was conducted in the absence of any commercial or financial relationships that could be construed as a potential conflict of interest.

## Footnotes

1 http://www.mothur.org

2 https://www.metaboanalyst.ca/MetaboAnalyst/home.xhtml
